# A Rare Case of Penile Strangulation by a Hard Plastic Bottleneck

**DOI:** 10.7759/cureus.10814

**Published:** 2020-10-05

**Authors:** Gorrepati Rohith, Souradeep Dutta, Sreenath G. S.

**Affiliations:** 1 General Surgery, Jawaharlal Institute of Postgraduate Medical Education and Research, Puducherry, IND

**Keywords:** penile strangulation

## Abstract

Penile strangulation is an uncommon condition that mandates emergent management to remove the constricting device and restoring penile blood flow. Different kinds of metallic and non-metallic objects were placed over the penis due to various reasons but mostly due to autoerotic intentions. We present a case of a middle-aged male who presented to the emergency with penile strangulation by a hard plastic bottle, which was successfully removed using a bone-cutter. The patient had an uneventful recovery without any immediate complications. Treatment aims at decompressing the penis to restore blood flow and maintain urethral continuity. Early removal of the constricting object with minimal discomfort to the patient prevents long term complications such as urethral stricture and priapism.

## Introduction

First described by Gauthier in 1755 [1], penile strangulation is an unusual surgical emergency arising in patients with paraphilic disorders, using various ring-shaped objects to extend the erection time and sexual gratification. Penile strangulation leads to a scenario similar to compartment syndrome with various degrees of vascular obstruction ranging from mild obstruction that resolves with decompression to complete gangrene of the penis. We present a case of a middle-aged male who presented to the emergency with penile strangulation by a hard plastic bottle, which was successfully removed using a bone-cutter.

## Case presentation

A 48-year-old gentleman was brought to the emergency with complaints of pain during micturition for the past four days. The patient was initially very hesitant in presenting himself before the doctors. On further probing by a team of doctors, he revealed that he had inserted his penis into a one-liter plastic bottle four days ago. He initially tried to remove the bottle using various lubricants such as soap, coconut oil, etc. but was unsuccessful. He cut most of the bottle in the process but found the bottle-neck too tight to remove. The patient refused to disclose the cause for the insertion of his penis into the bottle. He did not seek any medical care before the present consultation.

**Figure 1:**
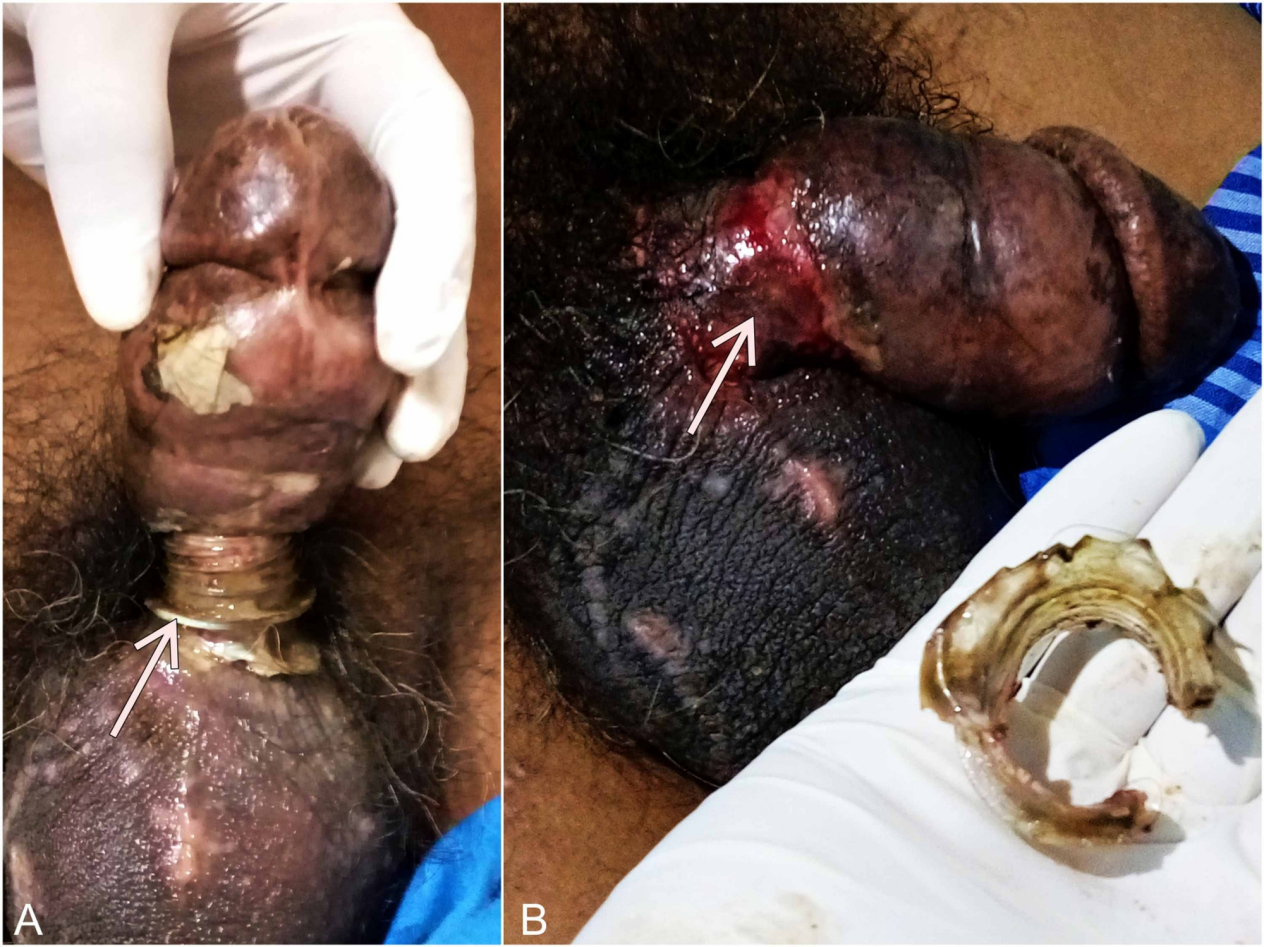
A: The hard bottle-neck at the root of penis (arrow). B: Ulceration seen at the root of the penis post constricting bottle-neck removal (arrow).

Clinical examination revealed a swollen and edematous penis with a constricting bottle-neck at the root of the penis (Figure 1).

There were multiple cut marks over his genital region that resulted during his attempts to cut the bottle-neck. The penis was cold to touch, and arterial pulsations were not felt due to the edema. Sensations were present over the distal penile region. The constricting plastic ring was successfully cut using a bone-cutter. After the removal of the constricting ring, the patient had immediate symptomatic relief. A doppler scan was done post-procedure which showed normal flow, and a retrograde urethrogram ruled out any urethral injury. The patient was continued on oral antibiotics for a period of five days along with local wound care, and he made an uneventful recovery. Psychiatric counseling was also provided to the patient following his recovery. 

## Discussion

Penile strangulation is a rare urological emergency that requires immediate removal of a constricting object. Objects such as nuts, bottles, rings in adults and threads, hair, or rubber bands in children have been previously reported for penile strangulation [2]. They tend to produce injuries of various grades ranging from mild penile engorgement to formation of fistula and gangrene. Injury is more pronounced in thin fibrous tissue covered corpora spongiosum and urethra compared to corpora cavernosa that is covered by thick tunica albuginea [3]. Metallic rings are comparatively easy to remove than non-metallic counter-parts, but the injury may be severe owing to their sharp nature. Acute urinary retention may occur, which might necessitate supra-pubic cystostomy (SPC). But the prognosis is better in these cases as the patient presents early.

Evaluating local temperature, color, sensation, distal pulsations, voiding ability are various ways to evaluate the case. Doppler scan can be employed in patients with absent pulsations. Different methods of removing constricting devices have been described, such as iron saw, dental drill, bone-cutters, etc.. If a drill is used, water should be continuously poured on it to thermal injury [4]. Aspiration of the corpus cavernosum was done to facilitate the removal. In severe cases, degloving of the penis to remove nuts and later to cover it with partial-thickness skin grafts were reported [3]. Care should be taken while removing the constricting device so as to prevent inadvertent injury to the penis.

Bhat et al. classified the penile strangulation injuries into five grades based on the extent of penile injury [3]: 

Grade I: Distal edema only

Grade II: Distal edema, skin and urethral trauma, corpus spongiosum compression, decreased penile sensation

Grade III: Skin and urethral trauma, no distal sensation

Grade IV: Separation of corpus spongiosum, urethral fistula, corpus cavernosum compression, no distal sensation

Grade V: Gangrene, necrosis, or distal penile amputation

As per this classification, our patient had a grade II injury and thus was conservatively managed. Patients with injuries of higher grades (grades III, IV, and V) should undergo SPC due to urethral injury [5]. Late complications such as urethral strictures and priapism may occur [3]. In the case of amputations, penile reimplantation using microsurgical techniques showed promising results [6]. In the long-term follow-up, investigations such as retrograde urethrography should be done to rule out urethral stricture.

Penile strangulation demands urgent intervention to avoid complications. Treatment should be aimed at removing the constricting device and restoration of penile blood-flow and urethral continuity. Psychiatric counseling will be helpful in preventing further such episodes.

## Conclusions

Penile strangulation is an unusual urological emergency arising in patients with paraphilic disorders using constricting devices around the penis. Treatment aims at decompressing the penis to restore blood flow and maintain urethral continuity. Early removal of the constricting object with minimal discomfort to the patient prevents long term complications such as urethral stricture and priapism.
